# A data-driven analysis of the potential of public transport for German commuters using accessibility indicators

**DOI:** 10.1186/s12544-021-00507-0

**Published:** 2021-09-26

**Authors:** Tudor Mocanu, Jigeeshu Joshi, Christian Winkler

**Affiliations:** Institute of Transport Research, German Aerospace Center (DLR), Rudower Chaussee 7, 12489 Berlin, Germany

**Keywords:** Commuting, Commuters, Public transport, Transit, Accessibility, Travel times, Mode choice, Mode shift, Open data

## Abstract

**Background:**

A significant mode shift will be required in order to meet the ambitious greenhouse gas emissions reduction targets in Germany and elsewhere. Such a mode shift can only be achieved by a combination of drastic push and pull measures. Getting commuters to switch modes might be particularly difficult and have a negative impact on their access to employment and welfare.

**Methodology:**

We investigate the potential for a mode shift from car to public transport for German commuters using a data-driven approach based mainly on open data sources that avoids complex transport model runs. Different datasets on the home and workplace location of all employees in Germany are consolidated to create an origin-destination commuter matrix at traffic analysis zone level. The commuter matrix is merged with travel time data for car and public transport to calculate a spatially disaggregated and mode-specific measure of accessibility. The comparison of accessibility by car and public transport is used to derive the potential for a mode shift and identify potential challenges and barriers.

**Results:**

Public transport accessibility to workplaces is poorer across the country compared to access by car. On average, public transport travel times are almost three times higher than the corresponding car travel times. The differences in accessibility are largely independent of the region type. Results are validated by an independent dataset from a household travel survey. Based on these results, the potential for a mode shift appears to be very low.

## Introduction

Commuting is an important segment of the transport market, both because of the relevance it has for the economy in a wider sense (enabling people to pursue economic activities), but also because of its sheer size. In Germany, about 20% of the total distance travelled by passengers stems from trips from and to the workplace, according to the latest national household travel survey [18]. Furthermore, facilitating travel to work locations is also high on the political agenda. In Germany, a tax allowance scheme (“Pendlerpauschale”) enables employees to deduct 30 cents per kilometer travelled to work from their income tax, thus incentivizing longer commuting distances [25]. This commuter allowance has even been increased for commutes longer than 20 kms as of January 2021, in order to compensate for the introduction of CO_2_ pricing (cf. [7] and [14]).

On the other hand, Germany, like most other developed countries, has set very ambitious targets in terms of reducing greenhouse gas emissions to be in line with the Paris Agreement [26]. However, German transport-related greenhouse gas emissions have been stagnating at around 160 million tons per year while the target for 2030 is 95 million tons per year [5]. A similar picture also emerges at EU level, where current trends do not yet point in the direction of the reduction targets set [9]. Reductions of greenhouse gas emissions of such magnitude can probably only be achieved by a combination of developments in the vehicle technologies (e.g. electrification) and mode shift and traffic avoidance measures.

For Germany, previous studies have shown that a sizeable mode shift can only be achieved by a combination of far-reaching and severe policy measures. For instance, Winkler and Mocanu [28] found that lowering the total distance travelled by car by 20% will require a policy package including both push (e.g. increase in fuel tax, road pricing, congestion charging) and pull measures (e.g. infrastructure upgrades for rail, public transport, cycling), using a model-based scenario analysis. Especially the push measures will likely have a large impact on the accessibility and welfare of the population.

This paper attempts to analyze the potential for a mode shift for commuters in Germany from a different perspective. Using a data-driven approach instead of transport models and scenario forecasts, this study does not focus on specific policy measures and their possible impacts. Instead, we analyze the status quo in terms of commuters’ mode choice and try to derive conclusions and learnings for the future from it. Building on information on the spatial travel patterns of commuters, we derive accessibility measures based on travel times. Liao et al. [15] recently presented a similar comparison of car and public transport travel times, though they do not focus on commuters and their specific travel patterns. In this analysis we intend to address two main research questions: (1) What is the difference in the accessibility of workplaces by car and public transport in terms of average travel times, and (2) What is the influence of the region type on the analysis results. The answers to these questions will help to highlight the potential for a mode shift and indicate possible barriers and challenges.

Beyond these aspects, the concept presented in this paper provides a data-driven approach that is mainly based on official statistics and open access datasets. The approach is therefore transferable to other regions and countries and could serve as a guideline for similar analyses for which no transport model is available or its usage is not appropriate.

The paper is structured as follows: in the data and methodology section we describe the data used for this analysis, how it was processed and also define an accessibility metric that forms the basis for the rest of the analysis. In the results section we present the main findings in terms of the commuters’ travel patterns and the accessibility of different regions and areas within Germany. In the discussion section we validate the results and draw the conclusions for the research questions formulated above. Finally, in the conclusion section we discuss some of the implications for future transport policy making.

## Data and methodology

### Overall approach

**Fig. 1:**
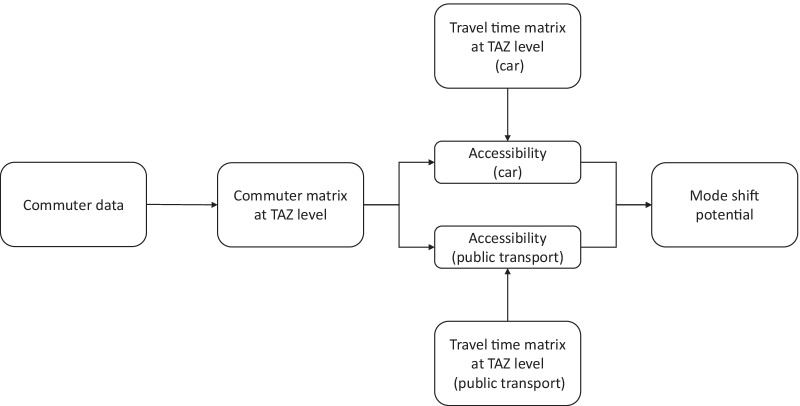
Flow diagram of the study methodology

The aim of this study is to evaluate the potential of a mode shift towards public transport for commuters in Germany using a data-driven approach based on the actual commuting relations and mode-specific travel times. The term “commuter” used here and in the remainder of this paper denotes all employees subject to social security contributions in Germany, irrespective of the distance between their home and workplace location. An overview of the methodological approach is shown in Fig. 1.

In a first step, various publicly available and commercial datasets on the Germany-wide commuting relations, home and work locations were processed and enhanced in order to maximize the spatial granularity and precision of the information contained therein. The result of the first step was a commuter matrix at traffic analysis zone (TAZ) level indicating the potential for commuting trips between the home and workplace location of all commuters considered. This matrix provides comprehensive information on potential trips from home to work within Germany and was the basis of the following analyses. However, it is important to note that this empirical matrix does not contain information on the actual trip frequency (if and how often commuting trips are undertaken), nor does it distinguish between different modes of transport.

In the second step, the commuter matrix was merged with travel time matrices for car and public transport derived from GTFS data and transport models. These matrices contain the estimated trip duration between the home and workplace location for both car and public transport modes. Finally, on the basis of the commuter matrix and the travel time matrices we derived an accessibility metric for both modes of transport and assessed the potential for a mode shift by comparing them.

### Commuter data

#### Data sources

The object of the following analyses are all commuters in Germany, irrespective of their home and workplace location (and of the distance between). However, in the official German statistics “commuters” are defined as employees subject to social security contributions whose place of work differs from their place of residence. Therefore, commuters with the same place of work and residence had to be derived from a different data source. Furthermore, the commuter matrix for this study was desired at the level of traffic analysis zones (TAZ) for the year 2019. Since the available commuter matrices were at different levels of spatial resolution and for different points in time, data processing was necessary to synergize the available datasets and obtain the desired dataset. Table 1 lists and briefly describes the data sources used to prepare the commuter matrix.

**Table 1:** Brief description and statistics of the data sources used in this study

Data source	Description	Spatial unit	Statistics
BfA [2]	Matrix of count of commuters subject to social security contributions by place of residence and work (year: 2019)	County (n = 401)	Commuters count = 13.0 million
BfA [1]	Matrix of count of commuters subject to social security contributions by place of residence and work (year: 2010)	Municipality (n = 11,748)	Commuters count = 16.3 million
Regionalstatistik [22]	Employed population subject to social security contributions at the place of residence (year: 2019)	County (n = 401)	Employed population = 33.2 million
Regionalstatistik [21]	Employed population subject to social security contributions at the place of residence (year: 2010)	Municipality (n = 11,748)	Employed population = 27.8 million
BKG [3]	Polygon features representing German municipal boundaries (years: 2010, 2019)	Municipality	No. of Municipalities: 2019 = 11,058; 2010 = 11,748
Nordenholz et al. [19]	Polygon features representing TAZ	TAZ (n =6633)	
Statistische Ämter des Bundes und der Länder [23], BKG v^4^]	INSPIRE based geographic grid system for Germany containing information on number of residences for each grid cell	Grid Cell size is 10000 sqm or 1 hectare	Total Population = 80.3 million

All datasets listed in the above table apart from BfA [1] (2010 commuter matrix by municipalities) are open and can be downloaded from the URL listed under References. The BfA [1] dataset is commercially available from Bundesagentur für Arbeit (German Federal Employment Agency).

#### Commuter matrix preparation

The entire process of preparing the desired commuter matrix was twofold. In the initial step the commuter matrix for the year 2019 at the municipality level was derived from the available commuter matrix at county level for the year 2019 and the commuter matrix at municipality level for the year 2010. In the next step the commuter matrix at municipality level was transformed to the TAZ level. The following sections describe the two steps in detail.

##### Deriving the commuter matrix for the year 2019 at municipality level

**Fig. 2:**
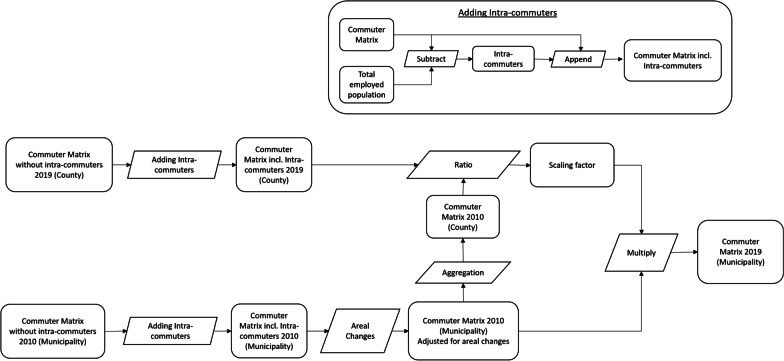
Flow diagram showing the steps taken to derive the commuter matrix for the year 2019 at municipality level

The commuter matrices do not include the employees whose place of work and residence are in the same county. These commuters (referred to as intra-commuters) had to be derived by subtracting the sum of all outgoing commuters (according to the commuter matrix) from the total number of employees living in each county and were then appended to the commuter matrix. The process of adding Intra-commuters is illustrated as a sub-figure in Fig. 2. This also explains why in Table 1 the commuter count at county level for the year 2019 (13 million) is lower than the commuter count at municipality level for the year 2010 (16.3 million).

The latest official commuter matrix for Germany was available for the year 2019. The areal unit of this matrix was county (n = 401). To enhance the precision of disaggregation of the commuter counts at municipality level for the year 2019 we used a commuter matrix for Germany from the year 2010 with a finer areal unit corresponding to municipalities (n = 11,748) [1]. Changes to the municipal boundaries and the consequent changes to the official municipal identification code were considered by calculating the weights for each municipality. The weights were calculated as the ratio of population that existed before the areal changes (i.e. for the year 2010) to the fraction of the population that remained in the municipality after the areal changes (i.e. for the year 2019).

Finally, a scaling factor was calculated to scale the commuter numbers from the year 2010 to match those from the year 2019. The commuter matrix from 2010 at municipality level, adjusted for areal changes, were aggregated at the county level. The scaling factor was then calculated as the ratio of the number of commuters between counties in 2019 to the number of commuters in the corresponding counties in 2010. The commuter matrix for the year 2019 at municipality level was obtained by multiplying the scaling factor with commuter numbers at municipality for the year 2010.

##### Transforming the commuter matrix to TAZ level

The commuter matrix derived from the official data sources has a spatial resolution that corresponds to the German municipalities. However, municipalities can vary significantly in terms of both area and population. For instance, the largest municipality in Germany, the city of Berlin, has a surface area of roughly 900 km^2^. For such large areal units, any analysis of travel times will likely be very imprecise. For this reason, we further disaggregated the commuter matrix to the level of traffic analysis zones (TAZ).

**Fig. 3:**
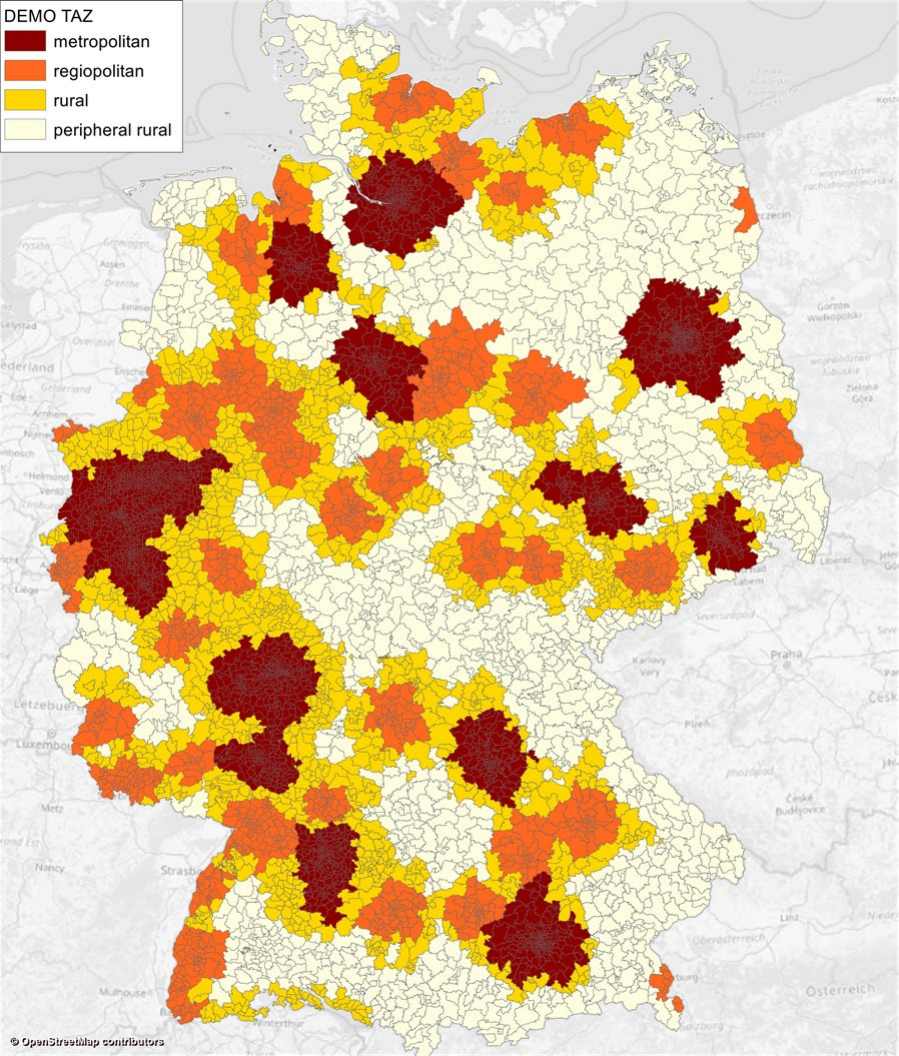
DEMO traffic analysis zones (TAZ)

The TAZ employed in this study were derived from the German National Transport Model DEMO. An in-depth description of the zoning system in DEMO is presented in Nordenholz et al. [19]. The territory of Germany is divided into 6633 TAZ of varying size, also considering the population and workplace density. TAZ in densely populated cities are smaller in size, whereas in sparsely populated rural areas they are larger and might encompass multiple (smaller) municipalities (Fig. 3). The TAZ are based on the municipal boundaries, with TAZ in urban areas being subdivisions of the cities and TAZ in rural areas being either one municipality or mergers of multiple ones. Other model-independent spatial zoning systems, such as an INSPIRE-based grid, can also be utilized for this step. The DEMO TAZ were preferred since their size is optimized to maximize the level of detail where needed while also limiting the total number of zones.

In order to disaggregate the municipalities-based commuter matrix, we used the weights of the TAZ given by their total home and workplace locations, while also factoring in a deterrence term based on the distance between the TAZ. The latter term was added in order to correct the distribution of commuting distances and somewhat “shorten” the trips within the municipalities.

From the final commuter matrix at TAZ level we removed those commuters with a commuting distance of over 100 kms. This threshold value refers to the beeline distance between the TAZ centroids. The main reason is that we have a focus on daily commuting trips. While some (few) commuters might have daily trips to and from work of more than 100 kms, these commuters are rather exceptions and it can be assumed that they have optimized their mode choice decisions very precisely. Furthermore, only 6% of all commuting trips fall in this category, but they are distributed over almost 90% of OD pairs. Considering them would make the analysis computationally very inefficient. For these reasons we only considered commuting trips of under 100 kms. In total, the final matrix at TAZ level consists of 31.2 million commuters.

### Travel time data

#### Car travel times

Travel times at TAZ level for the mode car were derived from the DEMO transport model. DEMO is a multi-modal, synthetic national transport model for Germany (cf. [28]). The supply side of DEMO contains a representation of the German road network consisting of all major inner and extra urban roads. In total, roughly 1 million links are included in the DEMO network model. Information on the link length, capacity and vehicle flow speeds under realistic traffic conditions are available for the entire network. For further information on the DEMO road network cf. Matthias et al. [16].

**Fig. 4:**
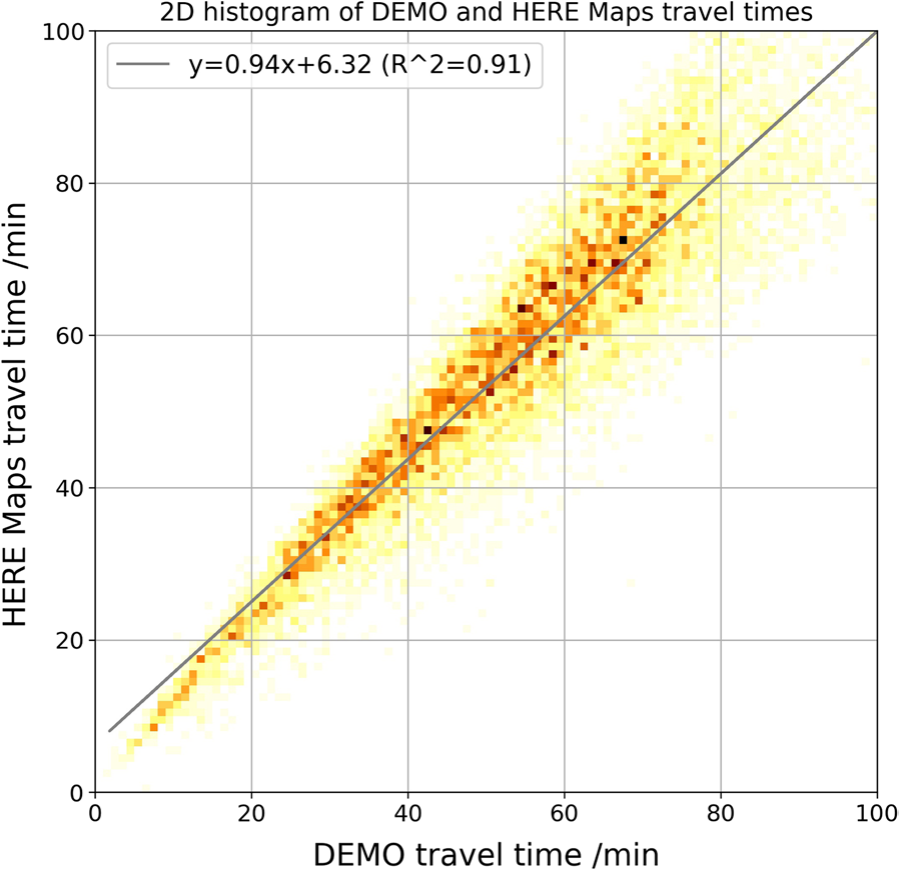
Comparison of DEMO and HERE Maps travel times

Travel times for TAZ origin/destination pairs were generated from the network by a series of shortest path searches under realistic traffic conditions. The total journey time (considered in this paper) consists of the in-vehicle travel time (sum of the travel time along the links in the network model) and the access and egress time to/from the vehicle parking location. The car travel times in DEMO were validated by comparison with the output of HERE Maps API queries. For this comparison, a sample of 10,000 TAZ to TAZ relations were queried from HERE Maps and compared to the DEMO travel times. A regression analysis revealed a coefficient of determination of $R^{2} = 0.91$ (see Fig. 4).

To generate travel time matrices, a transport model is not necessarily required. Other potential data sources for travel times include e.g. GIS data and navigation service providers, Application Programming Interfaces (APIs) from Google Maps or HERE Maps (cf. [27]) and, more recently, UBER Movement travel time data (cf. [29]).

#### Public transport travel times

Travel times for public transport were also derived from a transport supply model. The network representation of public transport is more complex than the road network, since it includes more than just links and nodes. Stop locations, transfer options, line routes, vehicle journeys and timetable information (departure and arrival times for each stop) are all required in order to generate accurate origin/destination travel times. Public transport timetable data is typically available from local transport operators or public authorities, who may publish it in a variety of formats, e.g. GTFS [12]. Obtaining a complete dataset for an entire country or larger region is particularly challenging, since data from different operators has to be acquired and merged into an integrated dataset.

**Fig. 5:**
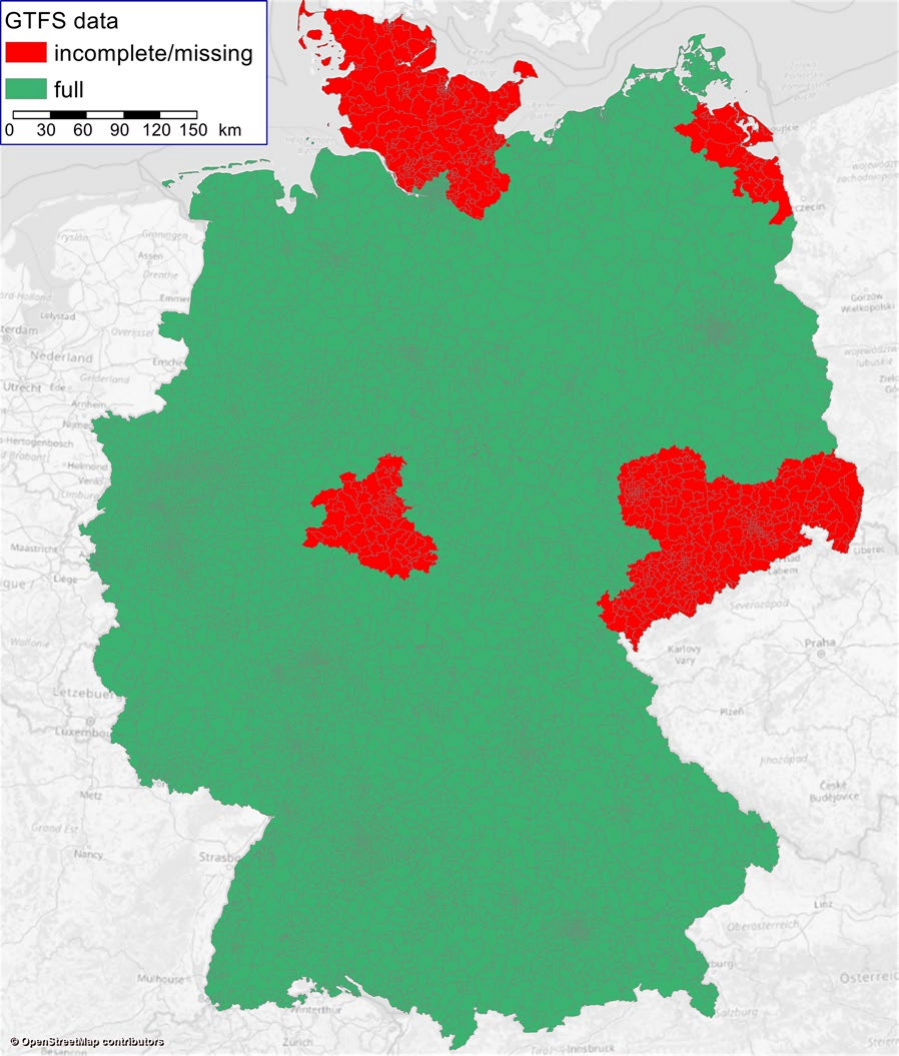
Availability of GTFS data in DELFI dataset

For Germany, a comprehensive open data source for public transport timetable data is provided by the DELFI initiative. DELFI regularly compiles and publishes a dataset including timetable data for the majority of rail and local public transport operators in Germany in GTFS format [8]. The DELFI dataset used for this analysis contains ca. 400,000 stops and 740,000 vehicle journeys per day from nearly 900 transport agencies. Timetable data is available for all public transport modes, including bus, tram, subway, light, suburban, regional and high-speed rail, ferry etc. As shown in Fig. 5, it covers most of Germany, with the exception of 34 counties (from a total of 401). A little under 3 million commuters (less than 10% of the national total) live in the area not covered by the DELFI dataset.

Utilizing the public transport travel times from the DEMO transport model was considered as an alternative to the DELFI GTFS dataset. This approach would have yielded the travel times for all TAZ and origin/destination (OD) pairs. However, the DEMO public transport travel times are the result of a complex model structure and thus are prone to a certain degree of imprecision. Since the percentage of population not covered by the DELFI GTFS data is relatively low, the impact of discarding these regions on the overall result was deemed lower than the impact of utilizing less precise data from DEMO. Consequentially, we relied solely on the DELFI data for this analysis.

From the GTFS network, travel times were derived using a complex route search and choice algorithm using PTV Visum transport modeling software (cf. [20]). The standard settings for public transport timetable-based assignment were utilized, with route choice being based on the perceived travel time (which also factors in the additional disutility from transfers and waiting times) and a Kirchhoff multi-route choice model. All public transport modes included in the DELFI dataset were considered. Travel times were calculated for the morning peak hours (7:00–9:00) of March 16, 2021, which was a regular (working) Tuesday. As with the car travel time, the total public transport journey times includes the in-vehicle time and the access and egress times, but additionally also considers transfer and waiting times.

Open source alternatives to derive travel times from GTFS data are also available, e.g. the OpenTripPlanner (cf. [30]).

### Accessibility indicators

Various definitions of accessibility are used in transport geography and spatial planning (e.g. [13] or [17]). Broadly speaking, accessibility is used to indicate how favorably-located an opportunity (e.g. workplace, commercial center etc.) is by assessing the impedances related to getting to that opportunity. Fayyaz et al. [10] give a very good overview and classification of different accessibility metrics. They find that the most common definitions are cumulative measures (number of opportunities/destinations reached within a given impedance) and gravity-based measures (weighting potential opportunities based on the trip impedance). These metrics are well-suited in a context where the actual destination of the travelers is unknown, i.e. where multiple destinations come into consideration. For the current study, this is not the case. If the corresponding home and work locations of commuters are known, cumulative and gravity-based accessibility measures become irrelevant.

We propose two different metrics of accessibility $A_{i,k}$ and $A_{j,k}$ that can be applied individually to each mode of transport and factor in the known origin and destinations of trips to evaluate the quality of commuters’ access to their work locations using that mode of transport. We define $A_{i,k}$ as

1 $$A_{i,k} = \frac{{\mathop \sum \nolimits_{j} T_{ij} \times tt_{ijk} }}{{\mathop \sum \nolimits_{j} T_{ij} }}$$

and $$A_{j,k}$$ as

2 $$A_{j,k} = \frac{{\mathop \sum \nolimits_{i} T_{ij} \times tt_{ijk} }}{{\mathop \sum \nolimits_{i} T_{ij} }}$$

where $A_{i,k}$, Hypothetical average travel time for all commuters with home in TAZ $i$ using mode $k$. $A_{j,k}$, Hypothetical average travel time for all commuters with workplace in TAZ $j$ using mode $k$. $T_{ij}$, Total commuters with home in TAZ $i$ and workplace in TAZ $j$. $tt_{ijk}$, Travel time between origin TAZ $i$ and destination TAZ $j$ using mode $k$.

Formulated as in Eqs. (1) and (2), $A_{i,k}$ and $A_{j,k}$ indicate the hypothetical weighted average travel time of commuting trips from and to a TAZ if all trips were carried out using mode $k$. Thus, the indicator is of a hypothetical nature since it is not based on real trips, i.e. it is not reflecting their day-to-day mode choices or how often the employees actually undertake the commuting trip. In other words, $A_{i,k}$ and $A_{j,k}$ represent the hypothetical usefulness of each mode of transport. However, since the exact commuting relations (given by $T_{ij}$) are used to derive $A_{i,k}$ and $A_{j,k}$, these indicators become a fairly accurate representation of the actual usefulness of each mode of transport in this specific choice context. Note that higher values of $A_{i,k}$ and $A_{j,k}$ indicate higher average travel times and thus a poorer accessibility, whereas for the more common cumulative and gravity-based accessibility metrics mentioned above it is the other way around.

The accessibility measures proposed for this study focus solely on travel times. Mode choice can also be influenced by other factors, such as costs, comfort, reliability etc. (e.g. [24]). Nevertheless, travel time remains the most important aspect and is readily comparable across modes, whereas other factors might be mode-specific and thus unsuitable for a cross-modal comparison.

## Results

### Commuting patterns

Before discussing travel time-based accessibility measures for car and public transport in Germany, it is reasonable to start with a brief look at the travel time- independent structural context and commuting patterns. Trip distances are influenced by several factors, one of which is the region type and corresponding density of employment opportunities and home locations. It is therefore necessary to analyze commuting patterns separately for different region types. For Germany, a suitable classification of region types in this context is the so-called RegioStaR classification (German: ‘Regionalstatistische Raumtypen’, English translation: ‘Regional Statistical Spatial Typology for Mobility and Transport Research) defined by the German Federal Ministry of Transport [6]. The objective of this classification is to delineate functionally homogeneous municipalities into spatial types. For all further analyses in this paper we chose the RegioStaR 4 categories, namely (1) metropolitan regions (2) regiopolitan regions (small and medium-sized cities) (3) rural regions (close to an urban region) and (4) peripheral rural regions (away from city regions). The four RegioStaR categories are also shown in Fig. 3. Note that there are no municipalities and no TAZ with more than one RegioStaR category, i.e., the borders between RegioStaR categories always follow municipal boundaries.

Table 2 gives the weighted average distance for outgoing and incoming trips for each of these region types. The outgoing trips are shorter for urban regions as compared to rural regions and vice versa for the incoming trips. This is in line with the theoretical expectations, that is (1) urban areas attract more commuters than their rural counterparts and (2) people in rural areas have to travel longer distances for the purpose of employment. Nonetheless, the differences in trip distances between urban and rural are not huge. A closer look at the numbers suggests that the weighted average distance for both outgoing and incoming trips for peripheral rural regions is lower than for rural regions. One possible explanation for this is that employees in peripheral regions are able to find home locations closer to their place of work.

**Table 2:** Weighted average outgoing and incoming trip distances for RegioStaR4 region types, in kilometers

RegioStaR 4	Outgoing	Incoming
Metropolitan region	14.98	16.67
Regiopolitan region	15.08	15.65
Rural region	17.61	14.82
Peripheral rural region	17.13	14.46

**Fig. 6:**
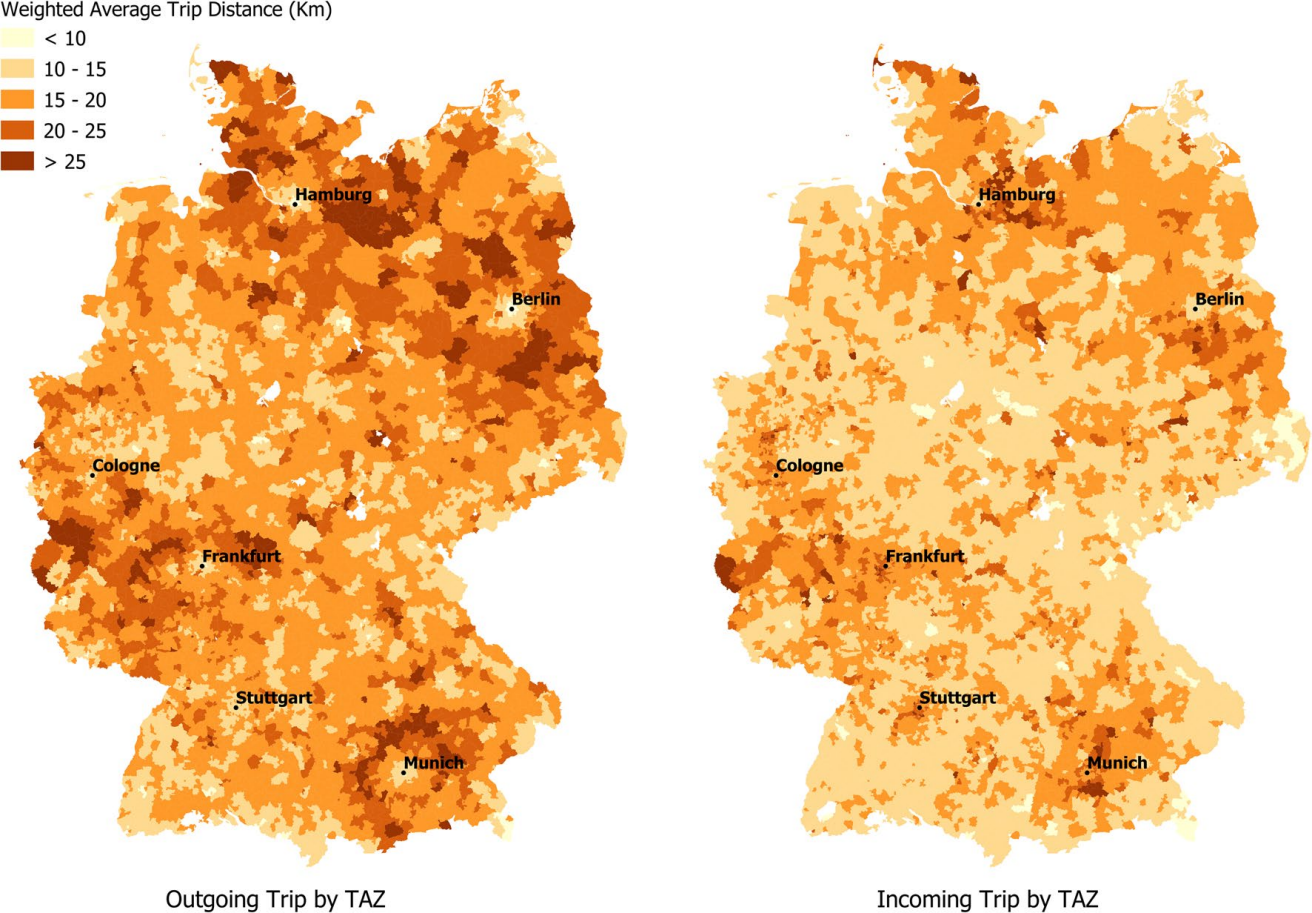
Weighted average trip distances in kilometers for **a** outgoing trips and **b** incoming trips by TAZ

**Fig. 7:**
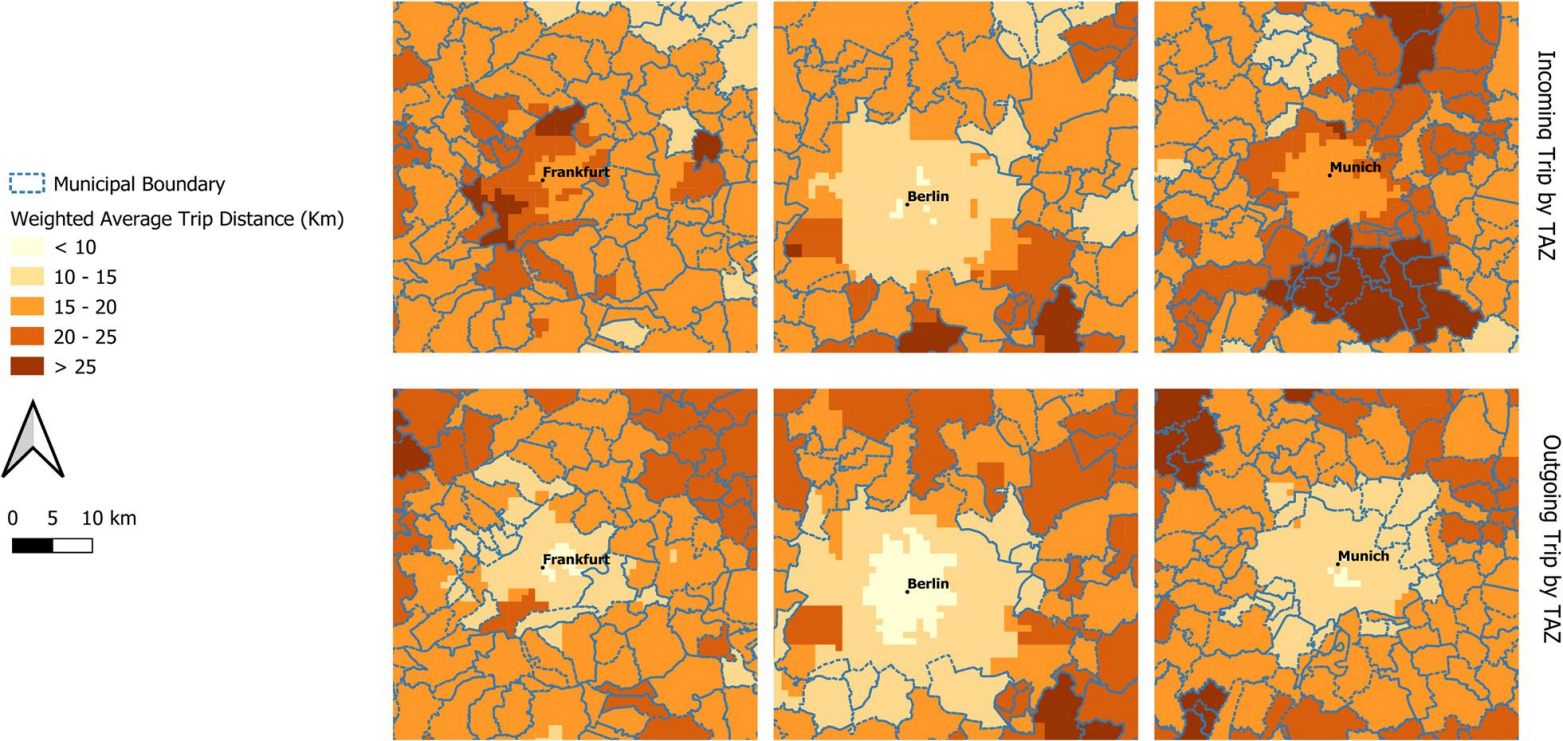
Comparison of weighted average trip distances for outgoing and incoming trips for selected metropolitan regions

Next, we explore the commuter patterns by TAZ. Figure 6 maps the weighted average trip distance for outgoing and incoming trips by TAZ. It is visible that the outgoing commuting distance is low for metropolitan regions like Berlin, Munich, Hamburg, Frankfurt etc., since most commuters living in these regions also work there. Conversely, trip distances are high for the sub-urban areas close to these metropoles, indicating that commuters living in sub-urban areas will more likely commute to the metropoles. Incoming trip distances for metropoles are a little higher that the outgoing distances (because of the longer distance incoming commuters), but are still relatively low, since most trips occur within the city. This is discernable in Fig. 7 which maps the weighted average trip distance at a larger scale (1:60,000) for selected metropolitan regions (Frankfurt, Berlin & Munich).

**Fig. 8:**
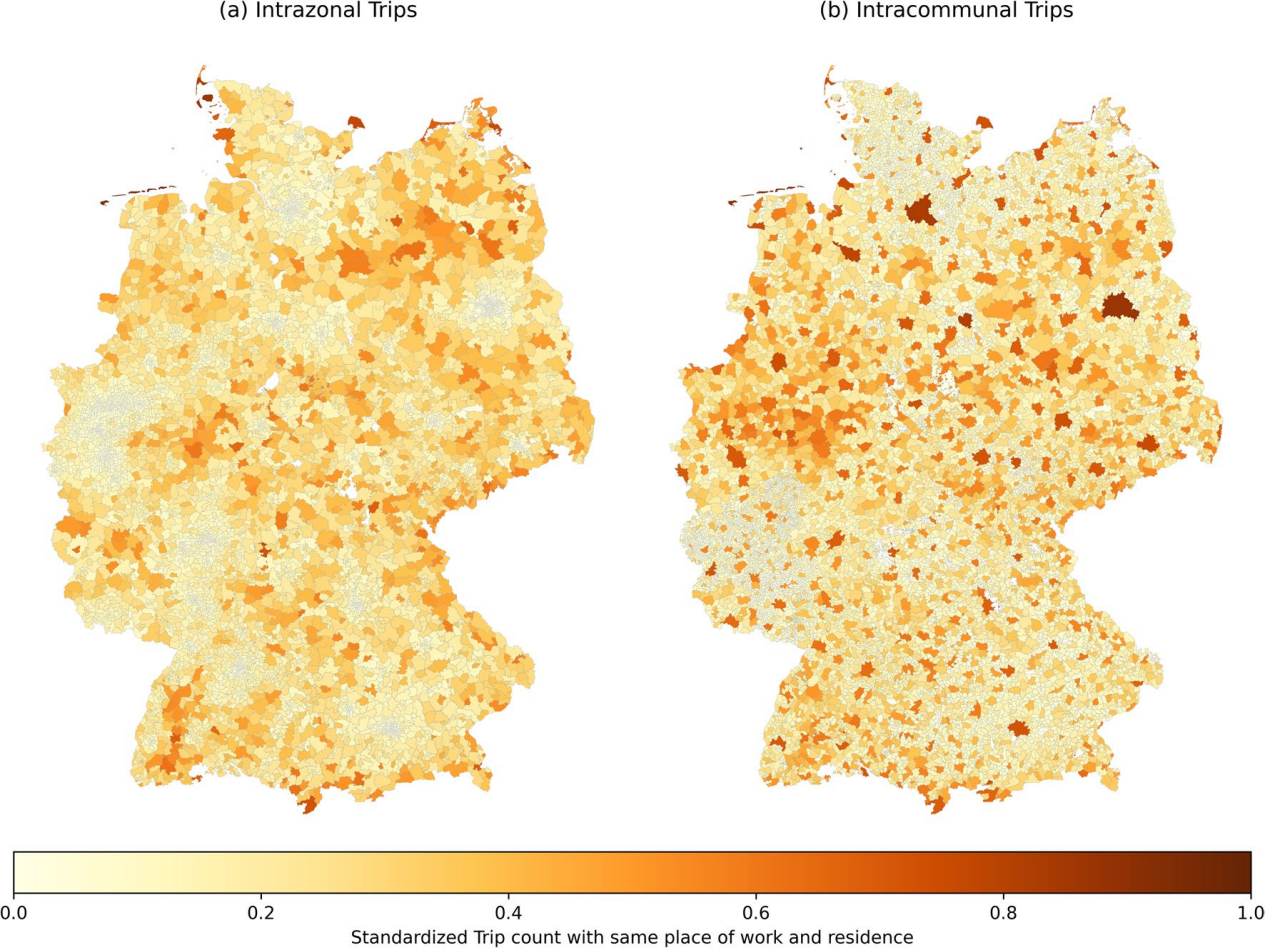
Standardized trip count with same place of work and residence by **a** TAZ and **b** municipality

Besides outgoing and incoming trips by TAZ, it is also interesting to study the pattern of commuters whose place of work and residence is the same (intra-commuters). As explained in the section describing the process of preparing the commuter matrix, these commuters have subsequently been added as they are not contained in the input commuter data. In total, there are 13.5 million intra-commuters at municipality level (commuters having their home and work locations inside the same municipality), which corresponds to about 40% of all commuters. In the largest German municipalities (e.g. Berlin, Hamburg), intra-commuters make up over 90% of all employees, as shown in Fig. 8b. After transforming the commuter matrix to TAZ level, only 5.5 million commuters have their home and work locations inside the same TAZ (see Fig. 8a). Note that intra-commuters at TAZ level are predominantly located in rural areas. For urban regions, the standardized trip count (percentage of intra-commuters from all commuters) is lower than average at TAZ level and higher at the level of municipality. This occurs because urban regions have finer grained TAZ (cf. [19]) and thus the probability of commuting inside the same TAZ is lower.

### Accessibility indicators (500)

**Fig. 9:**
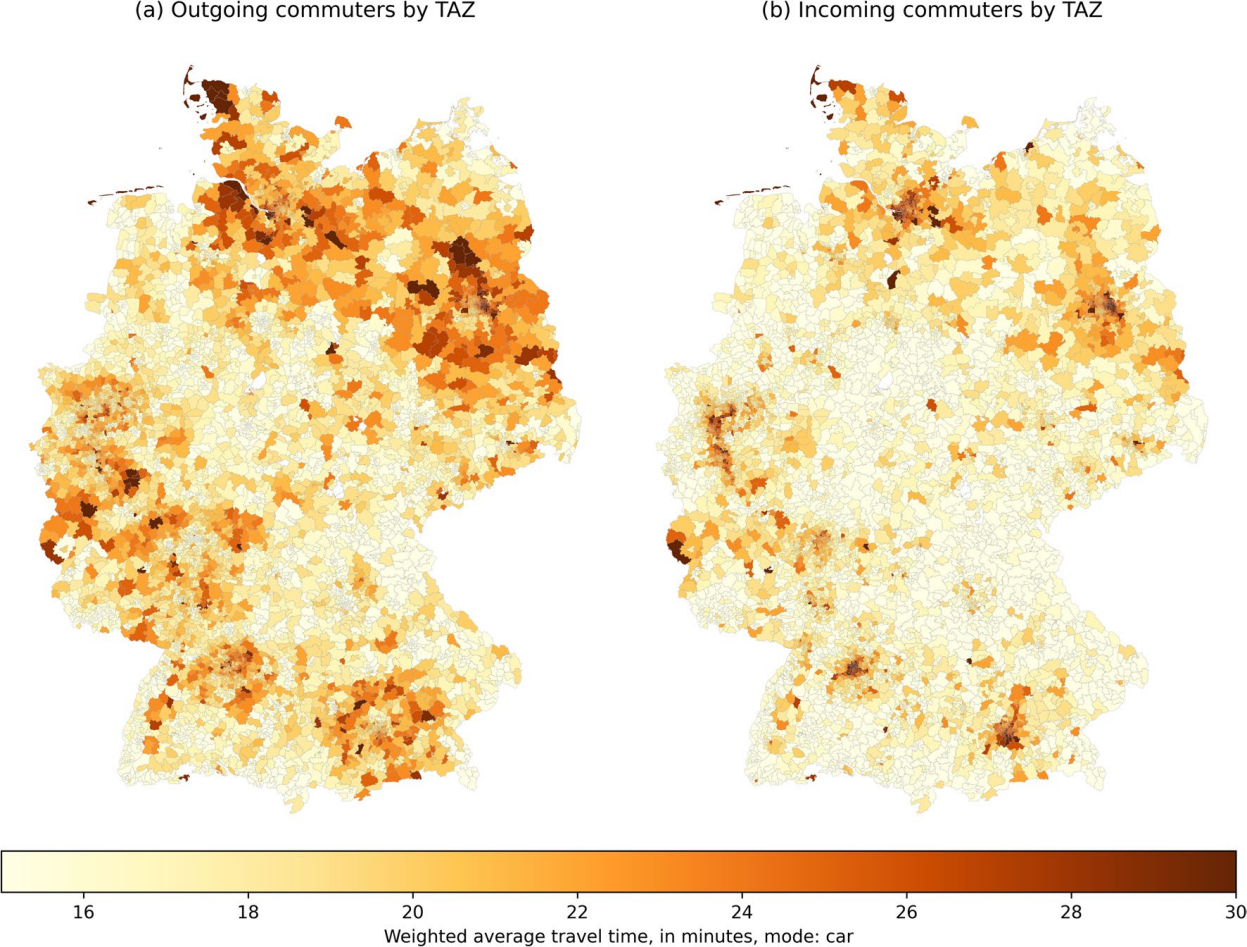
Hypothetical weighted average commuting travel times by TAZ, mode: car

The accessibility measures $A_{i,k}$ and $A_{j,k}$, indicating the hypothetical weighted average commuting times, were calculated at TAZ level for both car and public transport modes. The results for the mode car are displayed in Fig. 9 and closely resemble the average trip distance plots from Fig. 6. TAZ with the highest average travel times for outgoing commuters are located on the outskirts and regions surrounding the largest cities (Berlin, Hamburg, Munich, Cologne etc.). Conversely, the incoming travel time is highest for trips ending in the above-mentioned cities. These patterns are an indication that centrally-located TAZ have a high density of workplaces and attract many commuters not only from within the cities themselves, but also from the surrounding sub-urban areas, leading to partially congested roads and higher average travel times.

**Fig. 10:**
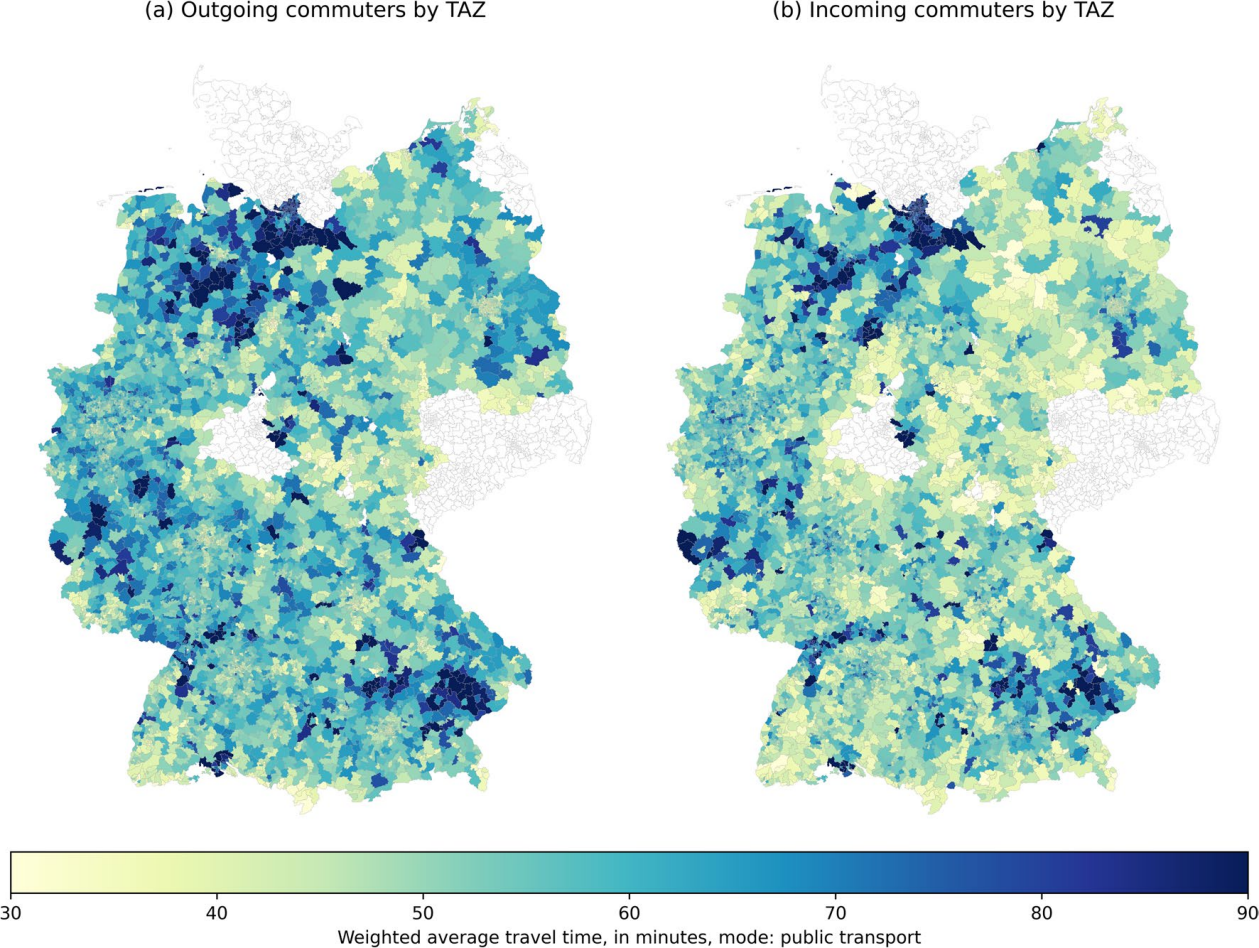
Hypothetical weighted average commuting travel times by TAZ, mode: public transport

In comparison, travel times by public transport are significantly higher across the country (Fig. 10). Note that the scale of the plot is different in order to display average travel times of up to 100 min per trip when using public transport. Also note that the regions with the highest average commuting times are not necessarily the same as those in Fig. 9. This indicates that for these regions, it is not only the higher commuting distances that lead to higher average travel times, but presumably also a lower quality public transport service. TAZ with incomplete/missing public transport travel time data are displayed in white.

For the purpose of this study, the main interest lies on the comparison of accessibility by car and public transport. The average travel times for both modes, grouped by the RegioStaR 4 categories, are shown in Table 3. Reaching the workplace by car takes, on average, between 15 and 25 min, depending on the region type. For public transport, the corresponding values are more than twice as high. This means e.g. that the average commuter living in a metropolitan area will need more than 30 min in addition per trip (one-way) if using public transport compared to using the car.

**Table 3:** Hypothetical weighted average commuting travel times by RegioStaR4 categories, in minutes

RegioStaR 4	Car		Public transport	
	$A_{i,car}$	$A_{j,car}$	$A_{i,pt}$	$A_{j,pt}$
Metropolitan	19.78	21.02	53.98	56.76
Regiopolitan	15.65	16.11	52.28	54.12
Rural	17.88	15.48	57.03	51.98
Peripheral rural	17.31	15.21	54.77	49.82

## Discussion

The accessibility indicators compiled in the previous section show very large differences in the travel times by car and public transport. Before analyzing these differences and their implications for a potential mode shift towards public transport, it is necessary to validate the results by comparing them to an independent data source. One possible data source could be mobile network data, from which travel patterns can be derived (cf. [11]). However, the spatial accuracy of mobile network data can be low, and, more importantly, separating the commuting trips from the rest of activities is not a trivial task.

Another option for validating the commuting travel times comes from travel survey data. The German national household travel survey Mobilität in Deutschland (MiD) [18] contains a record of nearly 80,000 commuting trips, including the trip distance, duration and mode of transport. Regional attributes (including the RegioStaR4 category) are also available for the home (but not the workplace) location of commuters. Note that the household travel survey only reports the (commuting) trips that the survey participants actually carried out on polling day, which might differ from their typical commute.

**Fig. 11:**
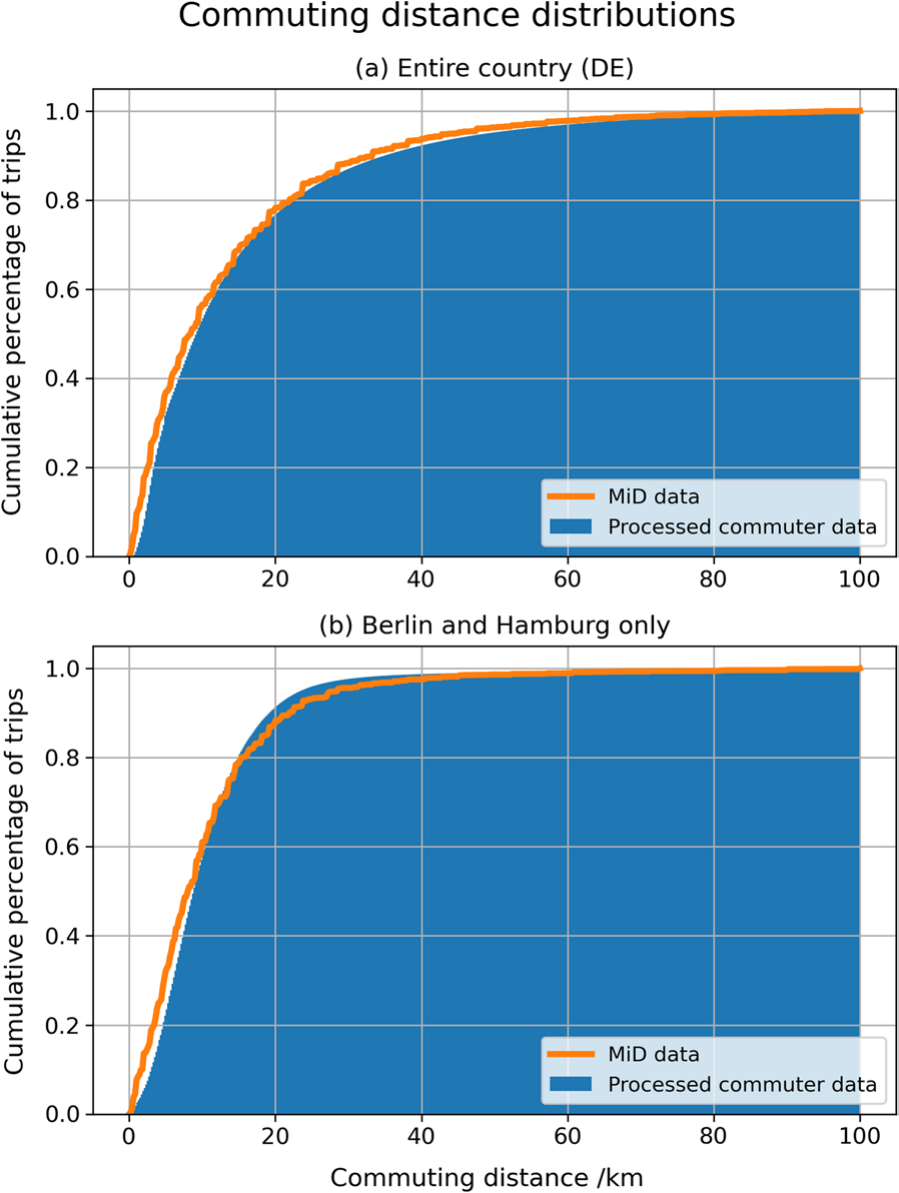
Comparison of commuting distance distributions between the processed commuter matrix and MiD

Although the commuting distance distribution results mainly from the commuter matrix input data, the process to disaggregate the flows to TAZ level might have an impact on the resulting trip distances, especially for the shorter trip distances inside metropolitan areas. Figure 11 compares the resulting commuter matrix at TAZ level with MiD data in terms of the cumulative distribution of the commuting distance for both the entire country and the two largest metropolitan areas (Berlin and Hamburg). Both charts show a very good fit between the MiD and the commuter data, validating the disaggregation process. Furthermore, Fig. 11 shows that trips in metropolitan areas are shorter than the national average, with around 90% of trips originating in Berlin and Hamburg being shorter than 20 kms, compared to less than 80% at national level for the same distance.

Since the household travel survey also reports the mode of transport chosen and the duration of each trip, it is also possible to compare the average travel times by car and public transport from the MiD with the accessibility indicators calculated of this study. It is important to repeat at this point that the accessibility indicator $A_{i,k}$ represents the hypothetical average travel time that would result if commuters would use mode $k$ for all trips, whereas the average travel times from MiD result from only those trips that were actually carried out with that mode.

Table 4 compares the outgoing travel times for car and public transport. For car trips, the reported travel times are higher than $A_{i,car}$ because in reality, the mode car is chosen less frequently for short trips (to the detriment of walking and cycling). If these short commuting trips were not considered, $A_{i,car}$ would increase by ca. 5 min per trip, making the results close to the reported trip times from MiD. However, short trips cannot be completely excluded from the analysis, since the sum of the mode shares of car and public transport reaches 50% even for trips shorter than 5 kms, according to MiD data.

**Table 4:** Comparison of $A_{i,k}$ with average commuting times from MiD by RegioStaR 4 categories, in minutes

RegioStaR 4	$A_{i,car}$	MiD (car)	$A_{i,pt}$	MiD (public transport)
Metropolitan	19.78	28.32	53.98	47.23
Regiopolitan	15.65	25.41	52.28	44.87
Rural	17.88	24.74	57.03	56.07
Peripheral rural	17.31	24.18	54.77	56.53

For public transport, this effect is also present, but it is offset by a different one. Public transport travel times are competitive only on few commuting relations where certain service quality conditions are met (e.g. direct connections, fast rail services etc.). These are the relations for which public transport has a higher probability of being chosen as the commuting mode. On other relations, where travel times are higher, public transport is presumably utilized less frequently. The latter relations are included in the calculation of $A_{i,pt}$ but are probably missing from the MiD trips (since public transport is not the chosen mode), which explains why the reported public transport trip durations are lower.

The results of the comparison of car and public transport travel times shown in Table 3 are also in line with those obtained by Liao et al. [15]. In their analysis of four cities (Sao Paulo, Stockholm, Sidney, Amsterdam) they found that public transport travel times were on average 1.4–2.6 times higher than the car travel times on the same relations.

After validating the results, two main findings can be derived from the analysis of the accessibility indicators. The first one is that there is a very large discrepancy between the accessibility by car and public transport. The hypothetical travel times are nearly three times higher by public transport than by car. The second main finding of this analysis, and potentially the more surprising one, is that the differences in accessibility of workplaces are largely independent of the type of region. Even for metropolitan areas, where public transport has a higher mode share, its service is of better quality and car traffic is potentially impacted by congestion issues, the differences in accessibility are still very large in favor of the car. However, it has to be noted that the travel time advantage of cars in dense urban areas can be somewhat diminished by considering the time lost while potentially searching for a parking space. It is not trivial to quantify these delays, since they can vary significantly by location (e.g. if there is a dedicated car park for employees or not) and time of day. Nevertheless, the difference between the travel times by car and public transport are so large that they cannot be offset by this factor.

The results presented above explain why in Germany the overall mode share of car is of over 60% for commuting trips, while only 15% of employees prefer public transport. Achieving a mode shift towards public transport will require to significantly lower the gap in accessibility by making public transport more competitive in terms of travel times. Under the premise of current service connection quality and travel times, the potential for a mode shift appears very limited and, if achieved with drastic push-measures, will severely impact commuters’ accessibility and welfare.

## Conclusions

The analysis presented in this paper attempts to shed a light on the potential for a commuting mode shift from car to public transport by comparing the accessibility of workplaces by the two modes of transport. Data on the home and workplace locations of commuters and on travel times by car and public transport was used in the analysis. The results show that accessibility by public transport is significantly lower for all region types and that for most commuters, public transport is not a competitive alternative in terms of travel times.

From a methodological point of view, the analysis is strongly based on available (open) datasets and avoids comprehensive transport model runs. Apart from the data manipulation procedures specifically required for the German commuter dataset, the approach could easily be adapted and applied to analyze mode shift potentials for other regions and countries as well. The application of this method is not restricted only to commuter data. Similar analyses could also be performed using other empirical (big) data sources on travel movement, such as mobile phone network or tracking data.

The findings of this study provide a valuable input to the discussion on possible changes in travel behavior of German commuters and how they could be fostered. Achieving a sizable mode shift would require either very strong “pull”-, very strong “push”-measures, or a combination of both. The obvious “pull”-measure would be to heavily invest in public transport and enhance the service quality to make this alternative competitive. The challenges associated with this path are manifold, including financial issues (heavy subsidies will likely be required to provide such service quality, particularly in rural areas), but also long and complex planning and execution processes for large infrastructure works. On the other hand, “push”-measures such as congestion charges or increasing parking fees might provoke a reaction from the commuters, but if they are not balanced by an improvement of the alternative modes they will lead to a reduction of the overall accessibility and welfare of commuters. A fine balance will be required in order to foster the acceptance and cooperation of commuters towards a mode shift.

The analysis presented here is intended as a starting point for a discussion on commuters’ (future) travel behavior and mode choice. Further research is required for a more in-depth analysis of several aspects. First, this analysis focuses on the travel times only. There are also other aspects that influence travel behavior and should be considered, such as costs, comfort, reliability but also intrinsic motivation and personal preferences. Second, there needs to be a better understanding of the future of commuting in general. Trends such as tele-working have been boosted by the recent Covid-19 pandemic, and it remains to be seen what the long-term consequences are. Third, the impact of vehicle automation and emerging mobility concepts is still unclear. Automation might help to increase the quality of public transport especially in rural areas, but it might also make car travel more attractive. Finally, this data-driven analysis should be complemented by model-based analyses of different scenarios on how to achieve the desired mode shift. However, for the reasons mentioned above these analyses should not leave out the questions of (individual) welfare, since these will be crucial in ensuring the acceptance of future pathways with drastic measures.

## Data Availability

Most of the input data used in this paper is open datasets which can be downloaded from the URL listed under References. All other datasets (with the exception of the 2010 commuter matrix, which was purchased from the source listed below) can be provided by the corresponding author to reviewers upon reasonable request.
